# Supplementary material for: The effect of increasing indoor ventilation on artificially generated aerosol particle counts
Source: PLoS One. 2021 Oct 8;16(10):e0258382. doi: 10.1371/journal.pone.0258382 (PMC8500404; doi:10.1371/journal.pone.0258382)
Supplement: S1 Data — (DOCX) [file pone.0258382.s001.docx]

| Group 1 |  |  |  |  |  |  |  |  |  |  |  |  |  |
| --- | --- | --- | --- | --- | --- | --- | --- | --- | --- | --- | --- | --- | --- |
| Date | Baseline | 0 mins | 5 mins | 30 mins | 60 mins | 90 mins | 120 mins | Temperature in F | Humidity % | Kitchen vent | Windows | Filter |  |
| 17-Nov | 178 | 9255 | 8344 | 3775 | 2550 | 1600 | 1232 | 70 | 40% | on | closed | off |  |
| 18-Nov | 204 | 23000 | 18343 | 6876 | 2908 | 1150 | 818 | 70 | 40% | on | closed | off |  |
| 1-Dec | 251 | 24334 | 15831 | 4030 | 1923 | 1212 | 853 | 70 | 40% | on | closed | off |  |
| 5-Dec | 193 | 19278 | 10388 | 4638 | 3075 | 2176 | 1173 | 70 | 39% | on | closed | off |  |
| 6-Dec | 309 | 16578 | 11345 | 4328 | 1967 | 987 | 789 | 70 | 37% | on | closed | off |  |
| 13-Dec | 179 | 19657 | 14567 | 5609 | 2980 | 1109 | 896 | 70 | 39% | on | closed | off |  |
| 14-Dec | 180 | 16547 | 14356 | 5609 | 2345 | 1203 | 902 | 70 | 38% | on | closed | off |  |
| 18-Dec | 296 | 12579 | 11900 | 2123 | 1810 | 1242 | 857 | 70 | 39% | on | closed | off |  |
| 14-Jan | 312 | 14003 | 12566 | 4123 | 3409 | 1921 | 1561 | 70 | 35% | on | closed | off |  |
| 17-Jan | 289 | 13455 | 12456 | 4021 | 2908 | 2100 | 1801 | 70 | 35% | on | closed | off |  |
| average | 239.1 | 16868.6 | 13009.6 | 4513.2 | 2587.5 | 1470 | 1088.2 |  |  |  |  |  |  |
| percent clearance | | |  | 100% | 35% | 20% | 11% | 8% |  |  |  |  |  |

Experimental raw data

| Group 2 |  |  |  |  |  |  |  |  |  |  |  |  |  |
| --- | --- | --- | --- | --- | --- | --- | --- | --- | --- | --- | --- | --- | --- |
| Date | Baseline | 0 mins | 5 mins | 30 mins | 60 mins | 90 mins | 120 mins | Temperature in F | Humidity % | Kitchen vent | Windows | Filter |  |
| 23-Nov | 283 | 13585 | 9679 | 7671 | 5460 | 4774 | 3998 | 70 | 39% | off | closed | off |  |
| 1-Dec | 258 | 14688 | 8528 | 6964 | 4569 | 3900 | 3143 | 70 | 40% | off | closed | off |  |
| 16-Dec | 250 | 13099 | 11545 | 9137 | 7519 | 4699 | 3415 | 70 | 35% | off | closed | off |  |
| 19-Dec | 301 | 13681 | 13833 | 7247 | 5200 | 3762 | 2501 | 70 | 36% | off | closed | off |  |
| 20-Dec | 135 | 15679 | 13456 | 11436 | 8567 | 6892 | 3789 | 70 | 37% | off | closed | off |  |
| 4-Jan | 367 | 16578 | 15988 | 13289 | 10287 | 8902 | 4096 | 70 | 40% | off | closed | off |  |
| 5-Jan | 279 | 17002 | 16010 | 14097 | 11090 | 8703 | 4002 | 70 | 38% | off | closed | off |  |
| 6-Jan | 231 | 15009 | 13256 | 10902 | 9002 | 7092 | 4145 | 70 | 36% | off | closed | off |  |
| 11-Jan | 382 | 12422 | 10842 | 7990 | 5974 | 4980 | 3419 | 70 | 35% | off | closed | off |  |
| 15-Jan | 214 | 15324 | 14561 | 7345 | 5102 | 4123 | 2305 | 70 | 35% | off | closed | off |  |
| average | 270 | 14706.7 | 12769.8 | 9607.8 | 7277 | 5782.7 | 3481.3 |  |  |  |  |  |  |
| percent clearance | | |  | 100% | 75% | 56% | 35% | 27% |  |  |  |  |  |

| Group 3 |  |  |  |  |  |  |  |  |  |  |  |  |  |
| --- | --- | --- | --- | --- | --- | --- | --- | --- | --- | --- | --- | --- | --- |
| Date | Baseline | 0 mins | 5 mins | 30 mins | 60 mins | 90 mins | 120 mins | Temperature in F | Humidity % | Kitchen vent | Windows | Filter |  |
| 21-Nov | 540 | 17869 | 10337 | 3311 | 2251 | 1852 | 1795 | 70 | 38% | on | open | off |  |
| 28-Nov | 705 | 30529 | 18366 | 4065 | 1966 | 1058 | 889 | 70 | 37% | on | open | off |  |
| 5-Dec | 237 | 15249 | 10637 | 4992 | 2358 | 1910 | 1120 | 70 | 40% | on | open | off |  |
| 8-Dec | 310 | 17890 | 12345 | 4576 | 2478 | 1568 | 986 | 70 | 39% | on | open | off |  |
| 15-Dec | 202 | 20552 | 13771 | 2964 | 1453 | 904 | 530 | 70 | 37% | on | open | off |  |
| 21-Dec | 223 | 17892 | 15467 | 6549 | 2908 | 1908 | 892 | 70 | 39% | on | open | off |  |
| 22-Dec | 239 | 15672 | 13459 | 6234 | 3021 | 1790 | 752 | 70 | 38% | on | open | off |  |
| 28-Dec | 310 | 16599 | 13672 | 6023 | 3201 | 1801 | 802 | 70 | 39% | on | open | off |  |
| 29-Dec | 206 | 17809 | 15409 | 5992 | 3002 | 1901 | 799 | 70 | 35% | on | open | off |  |
| 19-Jan | 234 | 18567 | 15677 | 3025 | 2545 | 1910 | 1110 | 70 F | 35% | on | open | off |  |
| average | 320.6 | 18862.8 | 13914 | 4773.1 | 2518.3 | 1660.2 | 967.5 |  |  |  |  |  |  |
| percent clearance | | |  | 100% | 34% | 18% | 11% | 6% |  |  |  |  |  |

| Group 4 |  |  |  |  |  |  |  |  |  |  |  |  |
| --- | --- | --- | --- | --- | --- | --- | --- | --- | --- | --- | --- | --- |
| Date | Baseline | 0 mins | 5 mins | 30 mins | 60 mins | 90 mins | 120 mins | Temperature in F | Humidity % | Kitchen vent | Windows | Filter |
| 7-Dec | 289 | 18732 | 11980 | 8756 | 6547 | 4231 | 3245 | 70 | 38% | off | open | off |
| 9-Dec | 289 | 16758 | 13452 | 8675 | 6653 | 4978 | 3617 | 70 | 40% | off | open | off |
| 10-Dec | 256 | 14567 | 13214 | 9806 | 7549 | 5078 | 3798 | 70 | 39% | off | open | off |
| 12-Dec | 251 | 15467 | 13298 | 7456 | 5435 | 3689 | 2345 | 70 | 37% | off | open | off |
| 13-Dec | 201 | 16789 | 14326 | 7809 | 5234 | 3678 | 2015 | 70 | 38% | off | open | off |
| 17-Dec | 428 | 10315 | 9246 | 6461 | 4160 | 3910 | 2100 | 70 | 36% | off | open | off |
| 12-Jan | 253 | 15248 | 14780 | 6357 | 5250 | 4464 | 2403 | 70 | 35% | off | open | off |
| 16-Jan | 350 | 13245 | 11987 | 6023 | 4765 | 3780 | 2510 | 70 | 35% | off | open | off |
| 18-Jan | 267 | 15436 | 13234 | 8803 | 7345 | 5023 | 2706 | 70 | 35% | off | open | off |
| 20-Jan | 310 | 16234 | 13412 | 9801 | 7609 | 5123 | 3102 | 70 | 35% | off | open | off |
| average | 289.4 | 15279.1 | 12892.9 | 7994.7 | 6054.7 | 4395.4 | 2784.1 |  |  |  |  |  |
| percent clearance |  |  | 100% | 62% | 46% | 34% | 21% |  |  |  |  |  |

| Group 5 |  |  |  |  |  |  |  |  |  |  |  |  |
| --- | --- | --- | --- | --- | --- | --- | --- | --- | --- | --- | --- | --- |
| Date | Baseline | 0 mins | 5 mins | 30 mins | 60 mins | 90 mins | 120 mins | Temperature in F | Humidity % | Kitchen vent | Windows | Filter |
| 19-Nov | 441 | 16263 | 7154 | 2864 | 1723 | 1200 | 1032 | 70 | 40% | off | Closed | on |
| 20-Nov | 250 | 23909 | 11088 | 5400 | 2128 | 1500 | 1122 | 70 | 40% | off | closed | on |
| 24-Nov | 225 | 17913 | 13961 | 3530 | 2991 | 1707 | 786 | 70 | 39% | off | closed | on |
| 11-Dec | 234 | 15302 | 13678 | 5678 | 3456 | 2031 | 1021 | 70 | 37% | off | closed | on |
| 12-Dec | 289 | 16578 | 12456 | 6543 | 3102 | 1908 | 987 | 70 | 36% | off | closed | on |
| average | 287.8 | 17993 | 11667.4 | 4803 | 2680 | 1669.2 | 989.6 |  |  |  |  |  |
| percent clearance |  |  | 100% | 41% | 22% | 14% | 8% |  |  |  |  |  |
|  |  |  |  |  |  |  |  |  |  |  |  |  |
