# Supplementary material for: The effect of increasing indoor ventilation on artificially generated aerosol particle counts
Source: PLoS One. 2021 Oct 8;16(10):e0258382. doi: 10.1371/journal.pone.0258382 (PMC8500404; doi:10.1371/journal.pone.0258382)
Supplement: Attachment — Submitted filename: Response to Reviewers.docx [file pone.0258382.s002.docx]

Ashwin Johri

Collegiate School,

103 N. Mooreland Road Richmond, VA 23229, USA

August 24, 2021

Dear Editor,

Thank you for your response and for providing the opportunity to resubmit my manuscript. The comments that I have received have definitely strengthened my manuscript and I am grateful for this input.

Please see below my point-by-point responses and related manuscript changes, and please let me know if these address your and the reviewers’ comments.

**Journal Requirements:**

1. *Please ensure that your manuscript meets PLOS One’s requirements, including those for file naming*

Response: The manuscript was modified as per the stated formatting guidelines

2. *In your Data Availability statement, you have not specified where the minimal data set underlying the results described in your manuscript can be found*

Response: A separate file with raw data set is uploaded under Supporting Information files

3. *Please upload a copy of Figure 4, to which you refer in your text on page 5.*

Response: The reference to Figure 4 has been removed

4. *We note that Figures 2 and 3 in your submission contain copyrighted images.*

Response: Figures 2 and 3 have been removed from the submission

5. *We note that you have included a table to which you do not refer in the text of your manuscript. Please ensure that you refer to Tables 1 and 2 in your text; if accepted, production will need this reference to link the reader to the table*

Response: Reference to tables 1 and 2 have been included in the text of the manuscript

**Reviewer’s comments to the Author**

Reviewer # 1

1. *In the first sentences of the introduction, the authors should update the figures on covid 19 (number of infection and deaths in the world)*

Response: The number of infections and deaths have been updated based on latest data Johns Hopkins COVID 19 coronavirus resource center from August 24, 2021

2. *In the introduction, I suggest to cite 3 studies:*

Response: The 3 recommended citations have been included as references 4, 11 and 20

3. *The authors should separate results from discussion*

Response: The results have been separated from the discussion

4. *In the discussion the authors should add something about practical implications of their experiment, studies limitations and short conclusion*

Response: The above mentioned segments have been separated and modified as per Reviewer 1’s suggestions. The practical implication of a simple method to improve indoor ventilation with kitchen exhaust vents has been included in the text. The limitations of the study which were included in the body of the text have been outlined with more clarity. A short conclusion has been added.

5. *More references are needed*

Response: The following references have been added in appropriate places in the manuscript: 3, 4, 5, 11, 13, 17, 19, 20

Please also note that in order to reformat and make the recommended changes in the context of the paper, other unrecommended changes were necessary. All changes are shown in the marked copy of the manuscript. The digital files were processed through Pace and corrected version is uploaded.

I would like to thank you again for your time and consideration and I would be happy to respond to any other queries should they arise.

Best regards,

Ashwin Johri
